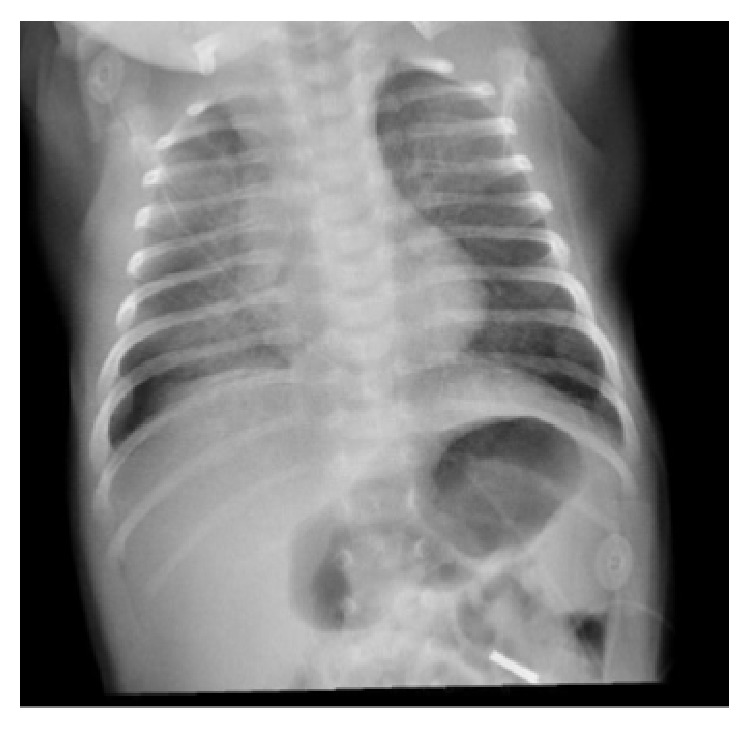# Neonatal Pulmonary Hemosiderosis

**DOI:** 10.1155/2014/463973

**Published:** 2014-10-19

**Authors:** Boris Limme, Ramona Nicolescu, Jean-Paul Misson

**Affiliations:** ^1^Department of Pediatrics, General Hospital Citadelle, Boulevard du 12 ème de Ligne 1, 4000 Liège, Belgium; ^2^Université de Liège, Place du 20 Août 7, 4000 Liège, Belgium

## Abstract

Idiopathic pulmonary hemosiderosis (IPH) is a rare complex entity characterized clinically by acute or recurrent episodes of hemoptysis secondary to diffuse alveolar hemorrhage. The radiographic features are variable, including diffuse alveolar-type infiltrates, and interstitial reticular and micronodular patterns. We describe a 3-week-old infant presenting with hemoptysis and moderate respiratory distress. Idiopathic pulmonary hemosiderosis was the first working diagnosis at the Emergency Department and was confirmed, 2 weeks later, by histological studies (bronchoalveolar lavage). The immunosuppressive therapy by 1 mg/kg/d prednisone was immediately started, the baby returned home on steroid therapy at a dose of 0,5 mg/kg/d. The diagnosis of idiopathic pulmonary hemosiderosis should be evocated at any age, even in the neonate, when the clinical presentation (hemoptysis and abnormal radiological chest images) is strongly suggestive.

## 1. Introduction

Idiopathic pulmonary hemosiderosis, a rare condition in newborns, can have both a rapid and dramatic clinical beginning, with pulmonary hemorrhage. A combined clinical and radiological approach is necessary to rapid diagnosis and therapeutic intervention, particularly in very young children in whom the pulmonary hemorrhage could be fatal.

The idiopathic feature keeps its actuality during neonatal period because the immunological mechanisms, supposed to be responsible for the disease, are rarely identified.

The immunosuppressive treatment should be started once the diagnosis is histologically confirmed. A long-term clinic and immunological follow-up is required, trying to make an etiological diagnosis.

## 2. Case Presentation

A 3-week-old previously healthy full-term newborn was presented to our tertiary hospital, shortly (15 min) after an episode of hemoptysis. No symptoms were reported by parents preceding the hemorrhage. There was no nasal cleaning and no trauma history. The hemoptysis was mistaken for nose bleeding with secondary laryngospasm.

The baby had been born at full term by normal vaginal delivery. There were no problems during pregnancy, neither at delivery nor at perinatal period. He was born to no consanguineous parents of Belgium descent. He has 2 female siblings both of whom are well and there is no family history of notable diseases. He had been well before this current illness, with appropriate neonatal evolution.

The baby is breastfed exclusively. Prevention of early and late vitamin K deficiency bleeding was assured by parenteral administration of vitamin K, according to national policy.

At his arrival in the Pediatric Emergency Department, on his pyjama, there was a lot of red blood. We have also traced the blood on his face, around the nose and inside of his mouth. On admission time, he was pale and hypotonic with moderate tachycardia (170/min) and moderate tachypnea (46/min). There was no fever. Central cyanosis (around the mouth) was noted. Initial oxygen saturation was around 96%, with progressive deterioration over the time. The capillary refill time was normal. There was no actively hemorrhagic skin or mucous lesions. The cardiovascular exam is normal, without clinical signs of congenital cardiopathy.

The pulmonary auscultation was also normal, and no alveolar crackles are identified. The abdominal examination reveals no palpable mass. The neurological exam and the archaic reflexes are normal. The remaining part of the systemic examination was unremarkable.

During his stay in the Emergency Department, a moderate grunting becomes noticeable and pink frothy sputum is visible on the lips. The oxygen saturation gets impaired (86%), so the baby requires some oxygen supplementation (3 L/min).

Initial routine laboratory work-up included a septic, haemostatic, renal, hepatic, and metabolic profile. All laboratory investigations returned normal, with 2 exceptions: moderate anemia (Hb 10 g/dL) and metabolic acidosis (pH 7,20, pCO_2_ 44 mm Hg, and bicarbonate 10 mmol/L).

A bolus of 10 mL/kg of saline serum was given, followed by glucose 5% with electrolytes perfusion.

First chest radiograph showed bilateral diffuse alveolar infiltrates over upper, middle, and lower zones (Figure 1).

The rhinoscopy procedure has found some red blood in the nose. There were no active hemorrhagic lesions.

The baby was admitted to the Intensive Care Unit (24 hours) under triple antibiotic coverage (followed up for only 24 hours, the time necessary to have back all bacteriological results, which were all negative). A progressive clinical improvement was noted and the newborn was discharged into the Pediatric Unit, where the investigational work-up was completed. There was no melenic stool. No other hemorrhagic episodes were noted during hospitalization.

Two thoracic scanners were performed and they were completely normal.

An immunological work-up (anti-DNA, antineutrophil cytoplasmic, and antiglomerular basement membranes and antinuclear antibodies) was performed and it was negative. Cow-milk allergy was ruled out.

Following the dynamic of siderophages generation in pulmonary alveoli (6, 7), a bronchoscopic alveolar lavage was performed on the 10th day in the evolution. Haemosiderin-laden macrophages (siderophages) > 98% were demonstrated in the bronchoalveolar lavage fluid (Golde score at 244). No vasculitis (interstitial neutrophilic predominant infiltration, fibrinoid necrosis of the alveolar and capillary walls, and leukocytoclasis) was described on histological result.

The immunosupressive therapy by 1 mg/kg/d prednisone was immediately started with a steroid taper to 0,5 mg/kg/d and the baby returned home on steroid therapy at a dose of 0,5 mg/kg/d.

The baby is regularly seen in external consultation and he is doing very well. Actually, the diagnosis is idiopathic/primary pulmonary hemosiderosis, but the infant's immunological profile will be regularly monitored over a long period for the early detection of any immunological abnormalities. A new, more extensive, immune check-up will be done at the age of 12 months.

## 3. Discussion

Idiopathic pulmonary hemosiderosis is an association of 3 key elements including recurrent episodes of hemoptysis, secondary, refractory iron deficiency anemia, diffuse alveolar infiltrates or opacities, and abnormal accumulation/presence of haemosiderin in the alveolar macrophages.

The incidence and prevalence of the disease in the pediatric population are still difficult to evaluate. To date there are around 500 cases reported in the literature [1].

The etiology and physiopathology remain also not well explained. Some etiologic hypotheses are debated, and for the pediatric age, the most cited are the allergic or autoimmune theories [1].

Age at the diagnosis is variable, with many cases presenting before the age of 10 [1, 2]. The evolution and therapeutic response are also individual characteristics.

The most frequent and classically described onset in childhood seems to be insidious, with recurrent hemoptysis secondary to diffuse or focal alveolar bleeding.

Secondarily, an extensive work-up for an unexplained, persistent iron deficiency anemia with typical chest radiographs, can also permit discovering a pulmonary hemosiderosis.

Less frequently, but much more dramatically, the first symptom is a moderate or massive pulmonary hemorrhage, potentially fatal [3].

The cause of IPH remains unknown, but an immunological origin has been suggested [2, 4]. Alveolar hemorrhage may be the first manifestation occurring long time (months to years) before the development of immunological disorders.

It was reported that some infants and young children with pulmonary haemosiderosis have plasma antibodies against cow milk proteins, and these patients dramatically improved on a cows-milk-free diet (Heiner's syndrome) [5].

Confirmatory diagnosis of IPH implies evidence of diffuse alveolar hemorrhage together with exclusion of other causes of pulmonary bleeding. In children of any age, some possible immune and nonimmune causes should be investigated and ruled out [5].

In acute hemorrhagic episodes, supportive management includes blood transfusions, oxygen therapy, high-dose corticosteroid therapy, or mechanical ventilation.

The long-term pharmaceutical therapy includes corticosteroids and when ineffective, other immune therapies should be envisaged.

## 4. Conclusion

We described here a case of neonatal pulmonary hemosiderosis, expressed clinically by an acute pulmonary hemorrhage with typical bilateral diffuse alveolar infiltrates and confirmed by the abundance of hemosiderin-laden macrophages in the bronchoalveolar lavage on the 10th day of the evolution.

## Figures and Tables

**Figure 1:**